# Three-dimensional resistivity and switching between correlated electronic states in 1T-TaS_2_

**DOI:** 10.1038/srep46048

**Published:** 2017-04-13

**Authors:** Damjan Svetin, Igor Vaskivskyi, Serguei Brazovskii, Dragan Mihailovic

**Affiliations:** 1 Jozef Stefan Institute, Jamova 39, 1000 Ljubljana, Slovenia; 2 Jozef Stefan International Postgraduate School, Jamova 39, 1000 Ljubljana, Slovenia; 3 CENN-Nanocenter, Jamova 39, 1000 Ljubljana, Slovenia; 4 Laboratory of Theoretical Physics and Statistical Models LPTMS–CNRS, UMR 8626, Université Paris-Sud Paris-Saclay, F-91405 Orsay, France

## Abstract

Recent demonstrations of controlled switching between different ordered macroscopic states by impulsive electromagnetic perturbations in complex materials have opened some fundamental questions on the mechanisms responsible for such remarkable behavior. Here we experimentally address the question of whether two-dimensional (2D) Mott physics can be responsible for unusual switching between states of different electronic order in the layered dichalcogenide 1T-TaS_2_, or it is a result of subtle inter-layer “orbitronic” re-ordering of its stacking structure. We report on in-plane (IP) and out-of-plane (OP) resistance switching by current-pulse injection at low temperatures. Elucidating the controversial theoretical predictions, we also report on measurements of the anisotropy of the electrical resistivity 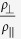 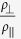
below room temperature. From the T-dependence of *ρ*_⊥_ and *ρ*_||_, we surmise that the resistivity is more consistent with collective motion than single particle diffusive or band-like transport. The relaxation dynamics of the metastable state for both IP and OP electron transport are seemingly governed by the same mesoscopic quantum re-ordering process. We conclude that 1T-TaS_2_ shows resistance switching arising from an interplay of both IP and OP correlations.

Layered transition metal 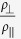
chalcogenides are attracting general interest as very versatile and multifunctional quasi-two-dimensional materials displaying competing charge density wave order (CDW), orbital order, superconductivity, and in some cases, magnetic order. 1T-TaS_2_ is of particular interest, as it is thought to satisfy the conditions for an unusual low-temperature Mott insulating state in which the electronic charge density within each layer is modulated by a three-directional IP CDW. This state may be described as a hexagonal array of polarons in the form of the star of David (Fig. 1a,c), defined by the Ta displacements towards the central charged Ta atom(Fig. 1a,b)^1^. The resulting low temperature (C) structure is commensurate with the underlying lattice (Fig. 1b,c), but spin ordering of the localized electrons at the polaron center is frustrated within the hexagonal lattice^2^, and no magnetic ordering has been reported so far. On heating above 220 K^3^ the commensurate state first breaks up into a striped polaron state around 220 K, and eventually to a patchy state of nearly commensurate (NC) domains separated by domain walls above 280 K. The material becomes superconducting under pressure^4^ or upon doping^5^,^6^,^7^. The electronic ordering perpendicular to the TaS_2_ layers and its role in determining out-of-plane (OP) transport and phase stability is currently highly controversial. Mechanically the material behaves as a 2-dimensional Van der Waals solid, with exfoliation properties similar to graphene, which at first sight suggests the inter-layer coupling to be weak. However, band structure calculations so far^8^,^9^,^10^,^11^,^12^,^13^ consistently predict insulating in-plane (IP) transport, with metallic out-of-plane (OP) transport. The latter is attributed to a highly dispersed band crossing the Fermi level along the OP, Γ–*A* direction in the Brillouin zone. The coherent OP transport in this band may be disrupted either by random interlayer stacking disorder possibly in combination with pair-wise layer stacking^10^ or helical disorder^14^, leading to Anderson localization and insulating resistivity behavior at low temperatures. In addition, the Coulomb repulsion between electrons on polaron sites are expected to lead to a Mott gap in the C state. The unusual duality in the predicted electronic transport and mechanical properties could be partly reconciled if we consider that transport relies on the overlap of *z*^2^ orbitals of Ta atoms on adjacent planes, while the mechanical properties depend on weak bonding by orbitals far from the Fermi level and Coulomb interactions between Ta and S layers^12^. However, in the absence of systematic transport measurements and electronic structure along the c axis, the issue of OP interactions, and indeed the importance of the IP correlations remain unresolved.

1T-TaS_2_ has recently attracted further attention because it was shown to exhibit sub-35 fs photo-induced^15^ resistance switching to a hidden (H) CDW state, with similar behavior induced by 40 ps electrical pulse-injection^16^. Recent reports of gate-tunable state switching to a supercooled NC state at low T^17^,^18^ and dynamical resistance switching^19^ are also indicative of the existence of multiple competing orders at low temperature. In the H state, the relaxation properties^20^ are strongly influenced by IP strain^21^, while sample thickness strongly influences the low-temperature electronic ordering^17^ confirming a strong susceptibility of the material to external perturbations along both IP and OP directions. Currently, the mechanism for switching, and the nature of the hidden state are hotly debated, which is closely linked to the controversial nature of the low-temperature commensurate ground state itself. In particular, the question is whether the pertinent physics is confined to the individual TaS_2_ layers, or three-dimensional stacking plays an important role in the switching and ordering^8^,^10^. Scanning tunneling microscope (STM) experiments also show switching by the tip, revealing the formation of domain walls (DWs) surrounding patches of a metallic phase in the a-b plane which is distinct from the surrounding (insulating) C state^22^,^23^. In neighboring layers, the nodes of DWs in one plane face domain centers on the neighboring planes, similar (but not the same) to that observed at higher temperatures in the NC state^24^. The implication is that inter-layer Coulomb interactions are not negligible.

In an attempt to resolve these questions, we address (i) the temperature dependence of OP transport, and (ii) the current-induced OP resistance switching with multi-contact measurements specifically designed for thin flakes. To elucidate the mechanism for switching, we also measure the switching dynamics and thermal relaxation properties of the metastable state in the OP direction and compare them with IP relaxation properties. The results are of fundamental importance for understanding the switching mechanism and also the OP correlations in the C ground state. On the practical side, we find that the observed c-axis switching opens the possibility of making thin film memristor devices in cross-bar geometry on polycrystalline thin films, or c-axis devices useful for ultrafast low-power low-temperature memories.

## Experimental Results

### c-axis resistance in the C state

While the IP resistivity 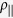 is easily measured by the conventional 4-probe method (see Methods), the c-axis resistance 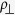 of very thin flakes cannot be measured this way. However, we can obtain 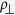 by combining 2-probe and 4-probe measurements on either face of the same flake with a multiple contact device shown in Fig. 2a and b (see Methods for details). Figure 2d shows 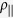 and 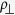 of a 75 nm thick flake of 1T-TaS_2_. We note a number of straightforward, yet important observations: The temperature-dependence of 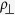 is qualitatively similar to 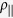 from 4 K to 300 K. While the magnitude of the anisotropy at intermediate temperatures 
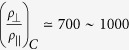
 shown in Fig. 2e is similar to that initially reported in this temperature range^25^, the data show a clear dip near the NC-C transition temperature *T*_*NC-C*_. The anisotropy then recovers to a relatively large value of ~1500 above *T*_*NC-C*_. Figure 2d also shows the c-axis resistance on a logarithmic plot, revealing a dominant component that was not discussed so far^26^,^27^: Thermally activated behaviour of the form 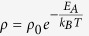
is seen between 40 and 140 K, with similar OP and IP activation energies of *E*_*A*_ = 112 ± 10 K and 91 ± 10 K respectively. Below 40 K, the Aarhenius plot (Fig. 2d) clearly shows that there is downturn in the resistivity with respect to the activated behavior. It’s known that *ρ*_||_ in this temperature range is sample dependent. Uchida *et al*.^28^ associated the presence of the upturn at low temperatures with higher quality samples and fewer impurities, which is consistent with the behavior reported upon Fe doping or substitution of S with Se^5^,^7^. The significance of the T-dependence data in the context of theory will be addressed in the Discussion.

### Resistance switching

This is performed with 2 contacts on either side of a 90 nm thick flake, as shown in the insert to Fig. 3a. The application of a 10 V, 50 *μ*s pulse at 20 K causes switching to a persistent low-resistance state. For comparison, the IP resistance switching is shown in Fig. 3b. Remarkably, the measured critical threshold current densities for switching are very similar: 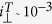 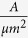 and 
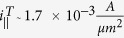
 respectively. Note that the value of the resistance *R*_*H*_ after switching is typically different than the extrapolated NC-state resistance, implying that the H state is distinct from the supercooled NC state reached by gating of very thin flakes in a FET configuration^17^. Performing the switching experiment on the device shown in Fig. 2, qualitatively the same behavior is observed for each opposite pair of contacts. Remarkably, we observe that each of the contact pairs 1–8, 2–7, 3–6 and 4–5 as shown in Fig. 2a,b, can be switched independently, implying that there is no IP cross-talk between adjacent contact pairs (or cells). The final state 2-contact resistance as a function of applied pulse amplitude is shown in the insert to Fig. 3b (Note that the contact resistance is not subtracted here). We see clear saturation behavior of *R*_*H*_ with two plateaus above 1 V, presumably corresponding to two different metastable states.

### Relaxation of the H state

The relaxation behavior of the H state resistance at different temperatures is summarized in Fig. 4. Above 35 K, the relaxation process is visible on a timescale of hours. The relaxation rate increases rapidly with increasing temperature, and above 50 K it is too fast to measure with the present apparatus. During the course of the relaxation, the resistance shows discrete jumps, in agreement with previous IP relaxation^20^. The OP relaxation curve is fit to a simple thermally activated law: 
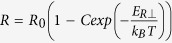
, with 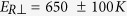 as shown in Fig. 4b, with the magnitude of *E*_*R*⊥_ similar to those obtained for IP relaxation on sapphire substrates^20^. (Here the error refers to the scatter between different samples). The difference between OP and IP relaxation is that the latter requires a stretch-exponential fit with a T-dependent exponent, while the OP relaxation does not.

### Dependence on pulse length and the role of heating

To determine the dependence of switching threshold on pulse length and to investigate thermal effects we perform pulsed measurements as shown in Fig. 5a. The resistance is measured before switching (R_0_) and at two times after switching (R_1_ and R_2_ at delays *τ*_1_ and *τ*_2_ respectively). Figure 5b shows the sample resistance (including contacts) as a function of time after a “write” (W) pulse for different *τ*_*W*_. Since switching to the H state is known to occur on a much faster timescale^16^, all we observe here is that for short pulses with *τ*_*W*_ < 200 *ms*, the system remains in the H state after the W pulse. We observe from Fig. 5b that the thermal time constants are of the order of tens of seconds, and the system relaxes to equilibrium after 100 s. R_2_ as a function of *τ*_*W*_ is shown in Fig. 5c (red squares) with *τ*_2_ = 250 *s*. We confirm that the switching resistance values are independent of pulse length up to 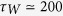 ms, in agreement with previously observed IP behavior^16^. With *τ*_*W*_ > 1 s, the application of a pulse no longer causes any resistance change as erasure by heating becomes important.

To determine the temperature to which the pulses heat the sample, and whence the role of heating in the switching mechanism, we rely on the characteristic R-T curves in the H state and C state in Fig. 2. Since heating causes “erasure” of the H state^16^, the resistance measured at short times can serve as a track record of the maximum temperature reached (T_max_), so we can determine the thermal cycle with reasonable precision. With a delay of *τ*_*1*_ = 1 s, *R*_1_ corresponds approximately to the maximum temperature reached during the pulse (crosses in Fig. 5c). With *τ*_*2*_ = 250 s the system cools to the base temperature (red squares in Fig. 5c). From Fig. 5c we see that the heating caused by short W pulses with *τ*_*W*_ ≤ 0.1 *s* is insufficient to raise the temperature by more than a few K. Thus, for *τ*_*W*_ < 0.1 *s*, 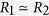, as the temperature never exceeds 40 K. With 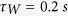, both R_1_ and R_2_ start to increase, the H state is partly erased, and the system no longer reverts to the initial state. With 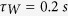, the measured *R*_1_ corresponds to a temperature *T* = 50 ± 5 *K*. With increasing pulse length, the difference between *R*_1_ and *R*_1_ becomes progressively larger. For *τ*_*E*_ ≥ 2 s, *R*_1_ indicates that *T* ~ 100 ± 5 *K*, and *R*_1_ approaches the C state value, indicating that the H state is almost fully erased, and the system reverts to the C state. We conclude that pulses with 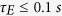 *do not* heat the system sufficiently to cause any thermal change of state, while thermal erasure is evident above 50 *K* in accordance with Fig. 2. Finally, we remark on the fact that although in principle the Joule heating can be up to 4 times larger in the C state than in the H state (because R is higher), a quadrupling of the heating with short pulses used here would still be far too small to cause *thermally*-induced dielectric breakdown. This confirms that the switching is electronic in origin as suggested previously for IP contacts^29^. Note the presence of intermediate metastable states with different resistances, depending on *τ*_*W*_, consistent with the proposed mechanism in ref. 29.

## Discussion

We first address the fundamental question of the IP and OP transport in the pristine (unswitched) state. The IP resistivity (Fig. 2c) shows a number of different regimes in different temperature ranges of the C state and is sample-dependent, so the following discussion should be understood in this context. The low-T behaviour has been attributed to either Anderson localization^26^, or a Mott gap splitting of the Ta 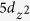 band at the Fermi level^30^. The presence of a clear gap in the density of states at the Fermi level seen by angle resolved photoemission^31^,^32^,^33^ and recent STM measurements is not consistent with either a simple Mott gap or Anderson localization. The observed behavior presented in Fig. 2 is also clearly not consistent with ordinary coherent OP band transport: the large observed anisotropy is quite in the opposite direction to what would be expected in that case. Its T-dependence (Fig. 2e) conveys some interesting information, however. Near the C-NC transition, the anisotropy strongly drops, showing a lag (hysteresis) between OP and IP behavior upon warming, implying that OP coherence is lost first, followed by the IP coherence. The more slowly varying anisotropy 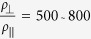 in the intermediate temperature range between 40 and 140 K is evident in the region where well-defined thermally activated behavior is observed. The relatively large resistivity anisotropy is 
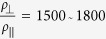
 in the NC state and it similar value in the C state implies that the intrinsic anisotropy for the electron transport is not directly linked to the IP ordering in either state.

In the NC state it was shown by Bovet *et al*.^12^ that columns of central Ta atoms with overlapping z^2^ orbitals give rise to half-filled 1D bands. In a strictly 1D half-filled band, the Mott insulator gap Δ_1D_ opens with no threshold, which is not true in higher dimensions, even for an arbitrarily small Coulomb repulsion U_1D_, irrespective of the bandwidth W_1D_. In 1T-TaS_2_ there is agreement that W_1D_ ~ 0.45 eV^8^,^9^,^10^. On the other hand, according to recent arguments^8^ the effective U_1D_ ~ 0.2 eV should be considered, rather than the much larger single-atom value taken in to emulate the Mott gap^11^. The resulting gap, roughly Δ_1D_ ∝ W_1D_ exp(−W_1D_/U_1D_), will compete with the small in-plane bandwidth W_2D_ ≈ 0.1 eV – and both can easily have comparable values. Another –diagonal – type of the inter-chain ordering was also recently considered^8^,^10^, with no inter-plane overlapping chains. In this case W_1D_ is reduced three times while W_2D_ is doubled. That would bring about a picture of a 3D narrow-band system where all bandwidths are comparable among themselves and with the Coulomb repulsion, so although the system is prone to the conventional Mott effect in the NC state, but there is no single dominant transport mechanism. ARPES shows gapless behavior near the Fermi energy, so there is no apparent inconsistency.

**Figure 1:**
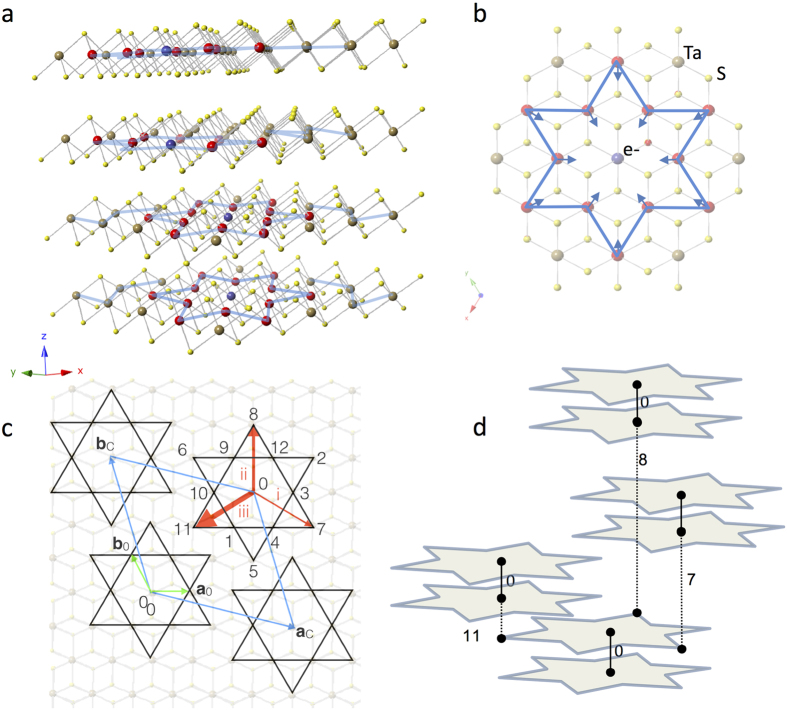
The charge density wave polaronic structure and interplane stacking in the commensurate phase of 1T-TaS_2_. (**a**) Each layer supports its own CDW, which may be viewed as a hexagonal array of polarons. The Ta atom at the centre of each polaron is colored blue, while the displaced Ta atoms are red. The extent of each polaron is schematically indicated by the blue line. (**b**) The detailed structure of the polaron showing atomic displacements. (**c**) Definition of the lattice and the CDW basis vectors within the commensurate in-plane structure and the lattice positions defining the stacking order according to ref. 14 (**d**) the C state helical layer stacking in the sequence 0-11-0-7-0-8-0… observed by Ishiguro and Sato^14^.

The situation is different in the C state where ARPES shows a large gap^9^. To try and better understand the *c*-axis transport in at low temperatures, let us consider the stacking structure in more detail. Full refinement of the structure from electron diffraction measurements by Ishiguro^14^ showed that the c-axis ordering in the C-state is formed by units of double 1T-TaS_2_ layers with overlapping David stars with the double layers shifted relative to each other by *a*_0_ − *b*_0_, *a*_0_ + 2*b*_0_ or −2*a*_0_ − *b*_0_ respectively as illustrated in Fig. 1d. Here *a*_0_ and *b*_0_ are the lattice constants of the undistorted lattice defined in Fig. 1c. With such helical stacking, the central Ta atoms are aligned within each double layer, but not between double layers, thus breaking the central Ta orbital hybridization register in the *z* direction. However, Ischiguro *et al*.^14^ also point out that defects in the stacking order are quite common, in overall agreement with X-ray^34^ and NMR data^35^, and the coherence length along the c axis is 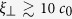
10,14. Phenomenological theory calculations of the stacking order by Nakanishi and Shiba^36^ confirm that double layer stacking has a minimum energy, but also indicate that an alternative stacking configurations may exist nearby, in agreement with the observed stacking disorder in the structural data. So far, we are not aware of any microscopic calculations which include the full helical OP stacking.

Ritschel *et al*.^10^ considered the simpler effect of two possible stackings **T**_s_ = 2**a**_**C**_ + **c**_**C**_ and **T**_s_ = **c**_**C**_, where **a**_**C**_ and **c**_**C**_ are the distorted unit cell vectors in the C state, considering random bi-layer stacking along the three equivalent vectors (2**a**_C_ + **c**_C_, 2**b**_C_ + **c**_C_ and −(**a**_C_ + **b**_C_) + **c**_C_). Their band structure calculations for **c**_**C**_ stacking predict an IP gap and metallic behavior along the *c* axis due to a single band crossing the Fermi level along the Γ − *A* direction. For 2**a**_C_ + **c**_C_ stacking, they predict metallic behavior in all three directions. Darancet *et al*.^8^ argue that the Coulomb interaction *U* for IP hopping is smaller than the c-axis bandwidth *W*_*C*_, suggesting that the Mott insulator picture of Fazekas and Tossatti^2^ can be valid for a single layer, but not for the bulk material. In this picture^8^, any insulating states in bulk material that do occur in the 1T-TaS_2_ family of materials may be regarded as arising more from OP antiferromagnetic order than from an IP Mott localization phenomenon.

**Figure 2:**
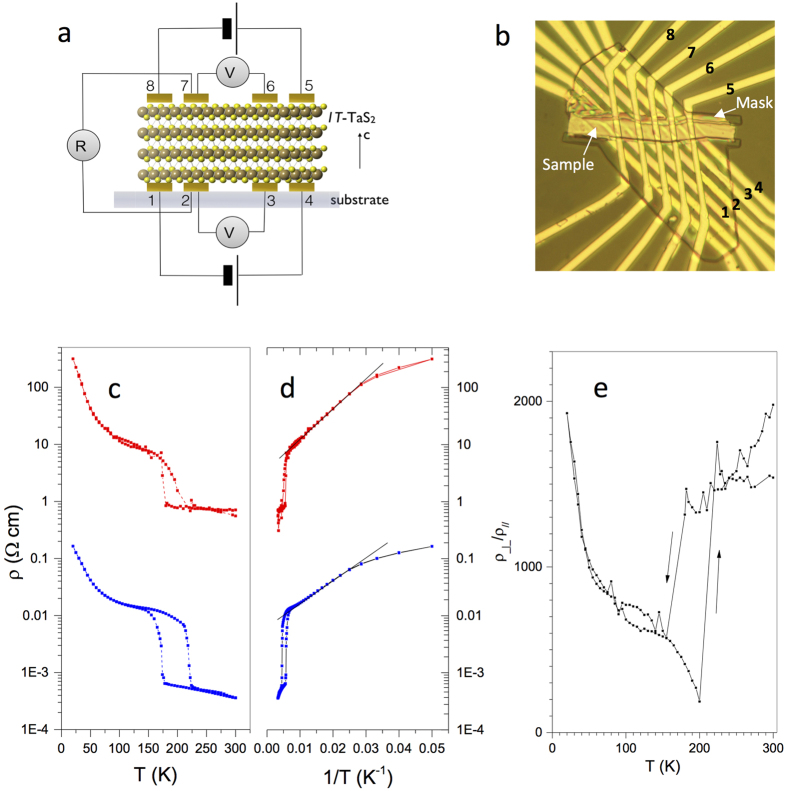
The in-plane and out-of-plane resistivity of 1T-TaS_2_ as a function of temperature measured on a multiple contact device. (**a**) A schematic diagram of the circuit, (**b**) a microscope image of the sample showing the contact configuration. The contact spacing is 10 *μm*. The masks (indicated) prevent unwanted currents through the side of the sample. (**c**) The in-plane resistance 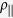 shows a 4-probe measurement (blue), while 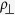 is a 2-contact measurement, with the contact resistance subtracted (red) (see Methods). (**b**) An Arrhenius plot of 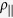 (blue) and 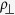 (red) revealing activated behavior over more than one order of magnitude of resistance between 40 K and 140 K. (**d**) The temperature dependence of the anisotropy of the resistivity 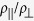 in the C state. The systematic errors are discussed in the Methods section.

Given that so far these predictions of microscopic theory are inconsistent with the data in Fig. 2, we need to discuss possible reasons. In ARPES and STM, the C and NC phases differ dramatically with the appearance of a gap in the C state, while the NC state is gapless. The sharp increase in 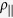 at 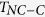 can thus be naturally attributed to the inhibition of low energy single particle excitations associated with the opening of a gap. However, in the region 40–140 K, the observed activation energy (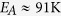 IP and 112 K OP) is far too small to be related to the Mott or CDW gaps whose values are at least 0.1 eV^37^, perhaps closer to 0.3 eV^10^. This energy scale 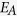 is not seen in any excitations which are measured by optical absorption, photoemission or tunneling. We note that these techniques detect individual single particle excitations, such as polaron binding energies or CDW gap excitations, but are not sensitive to collective polaron dynamics. The fact that the Arrhenius fit is so good over the temperature interval 40–140 K means that *E*_*A*_ is unlikely to be associated with random defects which would be expected to show a spread of activation energies. That also excludes the possibility that *E*_*A*_ corresponds to the energy difference between the Fermi level at *E*_*F*_ and the mobility edge *E*_*M*_ speaking against the Anderson localization mechanism for IP conduction in this temperature interval. Instead, the IP transport in this region is most likely related to collective polaron motion, where *E*_*A*_ corresponds to a well-defined transport barrier for polarons, such as may be imposed by domain walls.

Turning to the T-dependence of the OP transport, the remarkable similarity of the temperature-dependences of 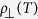 and 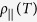 in all temperature regimes points towards a bottleneck mechanism which governs both IP and OP transport. OP conduction through interstitial impurities or even stacking “defects” is a very plausible mechanism for forming conducting links between layers. The OP transport thus reflects the IP mechanism, rather than being completely independent. Figure 2e shows that this is qualitatively consistent with the data over a large temperature range, except near the transition temperature *T*_*NC−C*_. We conclude the discussion of the theory in the pristine state by noting that so far the semi-phenomenological extension of the MacMillan theory of the incommensurability^36^,^38^ describes the 3D electronic ordering (including stacking) with surprising success, including the prediction of the 1^st^ order character of the phase transitions, something which so far eludes other more recent mesoscopic models. A more economical and robust approach considers domain walls and their crossings, which enables us to understand and model the IP switching under the optical^15^ pumping and, to an extent, the IP switching^29^. Further developments of microscopic theories are necessary to give a foundation for the phenomenology and derive the observable electronic properties beyond just structural information. As an outstanding problem, we note that no band structure calculations are able to reproduce the Mott insulator state. Still, the appearance of the insulating behavior in both IP and OP signifies that the emergence of the Mott state might require more sophisticated treatment than the DMFT-like calculations performed so far, at least considering the orbitronic effects of stacking. The observed behavior cannot be attributed solely to either IP ordering or OP double layer re-stacking, but indicates the existence of long-range-ordered 3D states in well-defined energy minima.

### The resistance switching mechanism

Generically, resistance switching in dielectrics and Mott insulators is discussed as a dielectric breakdown phenomenon. The question is what is the detailed mechanism in the case of 1T-TaS_2_. Considering that the largest change of lattice constant upon CDW re-ordering from the NC to the C state is in the c direction 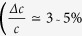, compared with IP change 
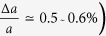
39, it is tempting to attribute the switching of the resistance to some kind of c-axis re-stacking. Following the calculations^10^, two metastable stackings of the orbitally ordered layers allow manipulation of salient features of the band structure, promoting the concept of controlling the properties of materials by using layer-stacking “orbitronics”. Indeed, competing stacking configurations were already indicated by the generalized Ginzburg-Landau-MacMillan theory^36^. The spin-unrestricted band calculations^8^ attribute the sensitivity of the metal-insulator phase boundary to the nature of the inter-plane *magnetic* ordering between the localized spin ½ electrons at the center of each polaron. This may lead to switching from OP antiferromagnetic - or even possibly ferromagnetic - reordering. So far the common feature of the band calculations which consider single or double layer physics is that metastabiliy in 1T-TaS_2_ is a consequence of »orbitronic« or stacking order, while IP order is robust and unperturbed according to these calculations

**Figure 3:**
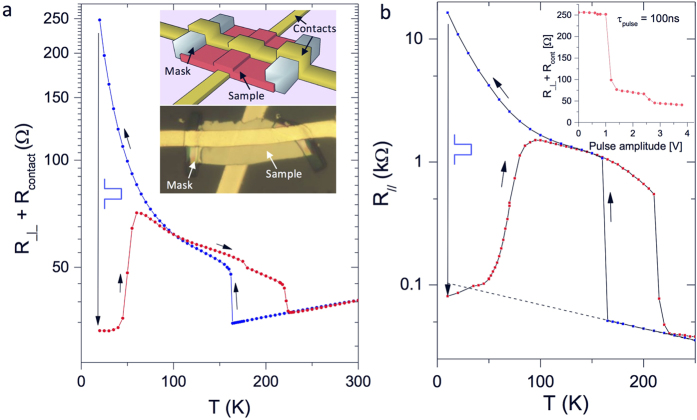
Resistance switching in 1T-TaS_2_ at 20 K. (**a**) The temperature dependence of the c-axis resistance (including contacts) before (blue) and after (red) the application of a 10 V 50 μs pulse. The insert shows the contact configuration and an image of the sample. The width of the contacts is 10 *μ*m. Note the mask at the edges, designed to prevent current leakage from the sides. (**b**) The temperature dependence of the in-plane resistance before (blue) and after (red) switching. The linearly extrapolated NC resistance is shown for reference. The insert shows the threshold for switching along the c axis with 100 ns pulses.

These theoretical notions are directly challenged by the observed anisotropy of the resistivity both in the pristine and the switched state (Figs 2 and 3). Moreover, recent STM studies of electrically switched 1T-TaS_2_^16^,^22^,^23^, clearly show that the IP reordering is govened by the appearance of a network of domain walls (DW). The switched IP textured state is very similar to the one originally predicted^15^ and is distinct from other states normally observed in equilibrium. Both the DWs and patches are metallic^16^,^22^,^23^, consistent with the conversion from polarons to itinerant states as proposed in^15^. The threshold current densities are quite similar for c-axis and IP switching: 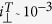 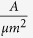
and 
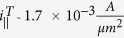
 respectively, while the applied threshold electric fields are vastly different for IP and OP switching (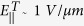 vs. 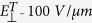). These observations are consistent with the original idea^16^ that the switching is driven by »saturable charge injection« in such a way that once a certain number of charges are injected, the domain pattern is formed and the transformation is complete. This also explains the independence of the threshold on pulse length shown in Fig. 5c^29^. Also, if the layers are considered equivalent, switching is a volume effect and the threshold current densities are expected to be the same, as observed.

**Figure 4:**
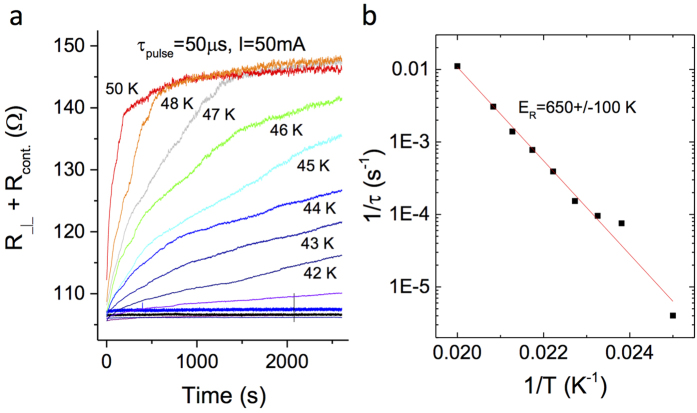
Relaxation of the H state resistance at different temperatures after switching with a 50 *μs* pulse (**a**). Note the steps superimposed on the smooth relaxation. (**b**) An Arrhenius plot of the activation energy *E*_*A*_ obtained from the fit to the resistivity relaxation data.

**Figure 5:**
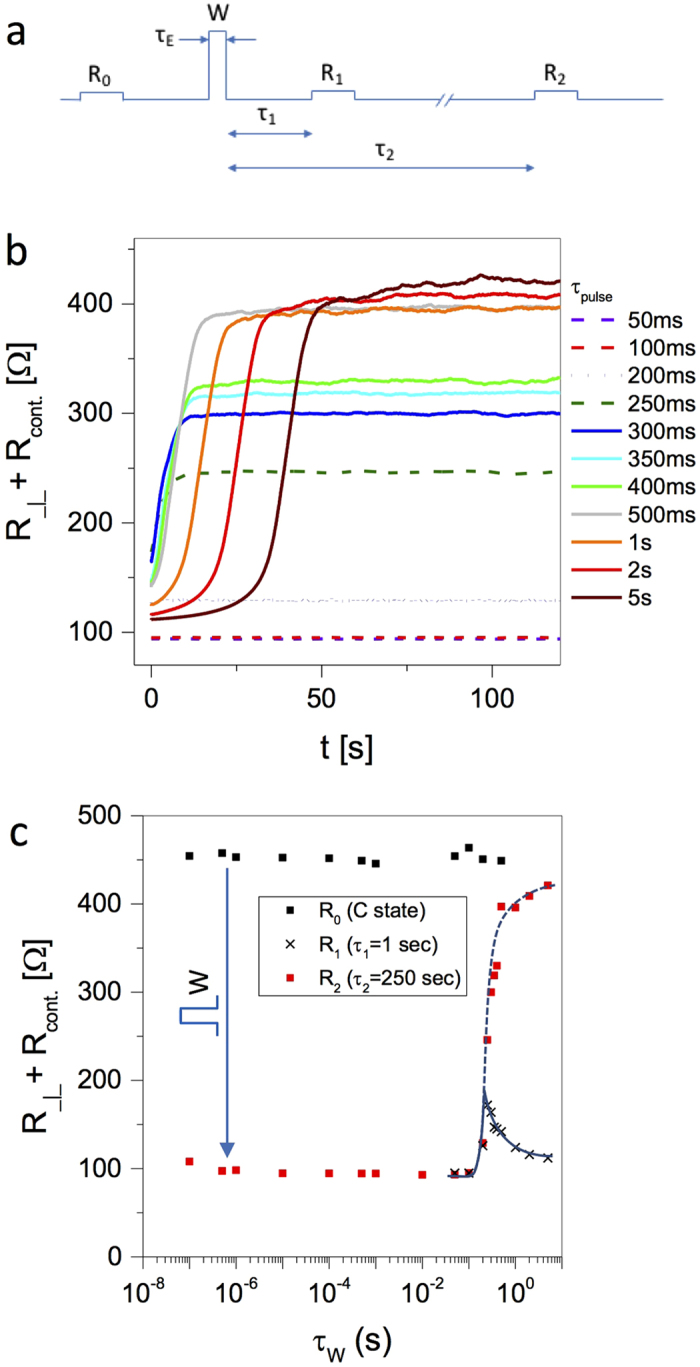
Resistance switching and erasure with different W pulse lengths τ_*W*_. (**a**) The pulse sequence used for the measurement of R_0_, R_1_ and R_2_. (**b**) Evolution of the sample resistance (incl. contacts) after W pulses of different length, as indicated. The thermal time constant is seen to be ~10 s. (**c**) The total OP resistance *R*_⊥_ + *R*_*cont*_ before (*R*_*0*_, black squares), and after the W pulse measured with *τ*_2_ = 250 s (*R*_2_, red squares) and *τ*_2_ = 1 s (crosses) for different *τ*_*W*_. The latter is a measure of sample heating. The sample is reset each time by heating to 300 K in between data points. The lines are guides to the eye.

The relaxation data of the switched state lend further support for the notion that both IP DW formation and re-stacking occur on switching: Fig. 4 shows nearly identical behaviour and a similar activation energy as previously reported for IP resistance^20^. This unambiguously implies that the IP and OP relaxation is governed by the same underlying processes. While the simple exponential fit to the time-evolution of the resistivity relaxation should be considered to be an approximation for describing the Ostwald ripenening nucleated growth process^40^,^41^, the fact that the IP relaxation process is better described with a stretch exponential fit^20^, indicates that the microscopic details of the OP relaxation are actually simpler than IP, with a single dominant energy scale given by *E*_*R*_. On the other hand, the distinct steps in the relaxation process are ascribed to the relaxation of complete rows of polarons at a time as recently observed by STM^16^. This scenario is consistent with the topological protection mechanism which was invoked to explain the stability of the H state at low temperatures^15^. The two processes together explain the observed smooth relaxation superimposed on quasi-random steps shown in Fig. 4a. Direct evidence for interlayer restacking after switching comes from STM images^22^, which show that the switched phase is accompanied by well-defined IP phase shifts of the CDW order parameter in the topmost layer, and by a phase shift of the CDW order parameter relative to the layer underneath.

### Switching applications with an all-electronic mechanism

Comparing the present system with other new materials showing memristive behavior currently attracting great attention, (for review see ref. 42 and the collection of articles in ref. 43 – and particularly the contribution^44^ therein) the mechanisms are quite different. Materials of choice are oxides, chalcogenides of transition metals^45^,^46^,^47^,^48^ and more complex compounds like AM4X8^49^. They are usually considered as systems with strong electronic correlations whose fingerprint is the Mott insulator state. There are also volatile cases of the bulk metal to (Mott) insulator transition, epitomized by VO_2_, see ref. 42 (and 50 for elucidating the local heating mechanism), but the single crystal degradation poses a problem. In most of these cases, the switching into metastable low-resistance configurations comes from non-volatile modifications, usually in local forms of filaments or granules, associated with the manipulation of ions by the current. There are exceptions from these mechanisms of “local switching” which are more relevant to our study: the work^51^ on Ca_2_RuO_4_ and the AM_4_X_8_ compounds^49^. There volatile, nonlocal, non-thermal switching effects have been found where the common feature seems to be electronic, rather than ionic ordering, but the phenomena are left unexplained so far. Our work, together with^29^, presents significant progress in comparison. Firstly, the conducting “hidden state” is truly stable within a large temperature interval. Second, the previously reported volatile switching times range from 400 ns^49^ to tens of msec i^51^, compared with switching between different charge-ordered electronic states in 1T-TaS_2_ which is exceptionally fast: record electrical switching speeds below 40 ps have been reported^29^, limited by external electronics. Finally, all-electronic switching means that the switching energy *E*_*B*_ may be extremely small. Sub-atto-joule/bit values may be achievable with small devices, assuming that the theoretically predicted scaling law holds^29^. The fact that adjacent cells in such a device shown in Fig. 1 can be switched independently without evident cross-talk implies that useful thin-film devices with unoriented films may be constructed in either cross-bar or lateral stripline geometry, opening flexible design options for new ultrafast low-temperature memristive memory devices based on CDW state switching.

## Methods

The samples were synthesized using the vapor phase transport method^26^. The devices are shown in Fig. 1a,b, and Fig. 2a. Their thicknesses were measured by atomic force microscopy and are 75 and 90 nm respectively. Thin flakes of single crystal 1T-TaS_2_ were placed over an array of contacts previously deposited on a sapphire substrate. To prevent IP current paths from the sides of the sample which might introduce an error in the measurement of the resistance perpendicular to the layers, an S8 polymer mask was then deposited at the edges of each contact as indicated. Finally, top gold contacts were made over the structure.

We cannot assume that the contact resistance is T-independent, or that the current paths are uniform between the contacts, so we need to subtract the relevant contact resistances at all temperatures without approximations. This can be done by placing 4 contacts on each side of the sample, and using a combination of 4-probe and 2-probe measurements as described in the following. Referring to Fig. 1, we name the contact resistances 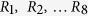, and the bare *sample* resistances between the contacts as 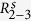, 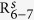, 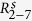, 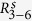. The standard 4-probe measurement using contacts 1-2-3-4 and 5-6-7-8, sourcing the current between 1 and 2 or 5 and 8, and measuring the voltage between 2 and 3 or 6 and 7 respectively, gives us 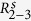 and 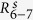 respectively. The 2-probe measurements between 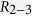 and 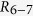 include the contact resistances such that 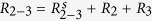, 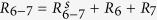, 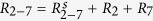 and 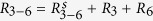. From these four equations we can calculate the sum of the c-axis resistances 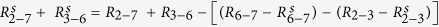. Assuming uniform sample thickness *t* and equal contact area *A*, the OP resistivity is then given by 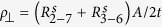 as plotted in Fig. 1c. The statistical error in the measurements of both IP and OP resistance is small and is indicated by the scatter of the points. Possible systematic errors for OP resistance introduced by the method are related to fact that the contact resistance on any particular contact may depend on the direction of the current, i.e. the current density distribution surrounding the actual contact for IP and OP current. This systematic error is hard to estimate, but is not likely to be more than a fraction of the contact resistance. Consequently, the qualitative picture will not be changed, and the greatest uncertainty will appear in the region where the sample resistance is smallest, i.e. 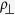 in the NC state, and the anisotropy data above 220 K (Fig. 2e), which is in any case not the main focus of this paper.

## Additional Information

**How to cite this article**: Svetin, D. *et al*. Three-dimensional resistivity and switching between correlated electronic states in 1T-TaS_2_. *Sci. Rep.* **7**, 46048; doi: 10.1038/srep46048 (2017).

**Publisher's note:** Springer Nature remains neutral with regard to jurisdictional claims in published maps and institutional affiliations.
